# Supplementary material for: Comparative Transcriptomic Analysis of mRNAs, miRNAs and lncRNAs in the Longissimus dorsi Muscles between Fat-Type and Lean-Type Pigs
Source: Biomolecules. 2022 Sep 13;12(9):1294. doi: 10.3390/biom12091294 (PMC9496231; doi:10.3390/biom12091294)
Supplement: Supplementary file 1 [file biomolecules-12-01294-s001.zip › biomolecules-1811104-supplementary.pdf]

Table S1. Primers for overexpression plasmid.

Gene	Primer	Sequence (5′-3′)	Annealing temperature (℃)
GALNT15-OE	Forward	CCCAAGCTTGCCACCATGGATGTTCGTAAGGAAGCGAGGC	65
	Reverse	CGGAATTCCCTTCTGACATGGCACTCA	65

Table S2. Sequence for siRNA.

Gene	Primer	Sequence (5′-3′)
Negative Control	Forward	UUCUCCGAACGUGUCACGUTT
	Reverse	ACGUGACACGUUCGGAGAATT
siRNA-1	Forward	GGAAGCUUUCCAGUAUUATT
	Reverse	UAAUACUGGAAAGUCUUCCTT
siRNA-2	Forward	GCCAUGGACAGACAUUACUTT
	Reverse	AGUAAUGUCUGUCCAUGGCTT
siRNA-3	Forward	GUCAGGAGAACAAUAAAGATT
	Reverse	UCUUUAUUGUUCUCCUGACTT

Table S3. Primers for RT-qPCR.

Gene	Primer	Sequence (5′-3′)	Annealing temperature (℃)
GAPDH	Forward	TCTTCTGGGTGGCAGTGAT	61
	Reverse	GTTTGTGATGGGCGTGAA	61
GOT1	Forward	CTGGATTCAAAGACATTCGG	59
	Reverse	AGGCTGAGTCAAAGAAGGG	59
RBP5	Forward	TGAACATCAACTTGGCTCTG	59
	Reverse	ATGGTCTGGCACTTTCGTC	59
XIRP1	Forward	AGAGAAGGTGATGGCAGAAC	58
	Reverse	GGAGGAACAGAGGCATTATG	58
CNN1	Forward	TCCTCTGCTCACTTCAACC	58
	Reverse	CTCGCAAAGAATGATGCC	58
HSPA6	Forward	CGCAGAATACCGTGTTTG	58
	Reverse	CAGTCTCCTTCATCTTGCTG	58
DAPK3	Forward	GCATTGCCCACAAGATTG	58
	Reverse	GAGGATGTAGGTGATGACGC	58
PENK	Forward	CCTTCTGTGGTTGACTTGATTG	58
	Reverse	CAGCATTTATGGGCGTTTG	58
MTFP1	Forward	GCTGATGCCATTGACAAAG	58
	Reverse	ATAGTGAAGCCTGGGATGC	58
PERP	Forward	GCTGTGGTGGAAGTGTTCTC	58
	Reverse	GCAGGAAGACAAGCATTTG	58
LOC100157061	Forward	GGAATCCAAGAACAATCAGCATCT	59
	Reverse	AGTGGGAGTCCTGGAACAAAG	59
LOC100622439	Forward	TCTCCTCGTATCTCAGTCTTGTTC	59
	Reverse	GTAAGGTGATAAGTGCTGCCATAG	59
ZNF648	Forward	CGAGCACCACGGAGATATTCAA	60
	Reverse	CAGAGTTCTTGTCCAGCCAGG	60
LOC106505263	Forward	TACGAGTTCACCTTGACGATGG	60
	Reverse	AAGTCAGGAGGCAGGTAGTTC	60
LOC110255980	Forward	TGGCAAGGGTCACTCTAGGA	59
	Reverse	GCTGGGGACCTTTCCATCTT	59
LOC100623270	Forward	TGTCACCCTGGAAATGGAACT	59
	Reverse	TGTAATGTGCTTGTCTGCGTAAC	59
LOC106505720	Forward	GAGGTTCTGCTTTCCAGTGTCTTA	60
	Reverse	GCCACGCTTACCTTCTTGTTATTG	60
LOC110255759	Forward	CTGCTGATGGACTCTGGGTTC	60
	Reverse	CCTCTTCTGCTGGCTCTCTTT	60
LOC110257277	Forward	TCCTCCAACTCCCATTCTTCATT	59
	Reverse	CCTCAGCAACAACACACCAAT	59
LOC110260256	Forward	GGCTGGAGGGAGAGATTGAAATG	61

	Reverse	CTGCTTGGTTCTGCGTTGTTG	61
ssc-miR299-5p		TTACTACACCCGTCCCGTGC	60
ssc-miR397-5p		CAACTATTCAGGGCGGAGCAG	60
ssc-miR1156-5p		ACTCATGAGTCCACCTGCCG	60
ssc-miR204-5p		AACACGCTCTCTCCAGCCTC	60
ssc-miR190-3p		AGTATTCTGTGCTGCTCGGGT	60
ssc-let-7a		CAGCGCTGAGGTAGTAGGTTGTATAGT	60
ssc-miR1358-5p		TCAGCTTCGGGCCTAGATCCT	60
ssc-miR1379-3p		CTGGGATCTCTGGGTTTGGGC	60
ssc-miR1379-5p		TGTGCCAGTGAAACCAGGTCT	60
ssc-miR356-5p		TATGTTGGGTCTGGGGTGTGT	60
ssc-miR573-5p		TATCGTTGGGTCTGGGGTGT	60
ssc-miR-10383		GTAACCTGACGTCTTGGCAGT	60
